# SARS-CoV-2 detection status associates with bacterial community composition in patients and the hospital environment

**DOI:** 10.1186/s40168-021-01083-0

**Published:** 2021-06-08

**Authors:** Clarisse Marotz, Pedro Belda-Ferre, Farhana Ali, Promi Das, Shi Huang, Kalen Cantrell, Lingjing Jiang, Cameron Martino, Rachel E. Diner, Gibraan Rahman, Daniel McDonald, George Armstrong, Sho Kodera, Sonya Donato, Gertrude Ecklu-Mensah, Neil Gottel, Mariana C. Salas Garcia, Leslie Y. Chiang, Rodolfo A. Salido, Justin P. Shaffer, Mac Kenzie Bryant, Karenina Sanders, Greg Humphrey, Gail Ackermann, Niina Haiminen, Kristen L. Beck, Ho-Cheol Kim, Anna Paola Carrieri, Laxmi Parida, Yoshiki Vázquez-Baeza, Francesca J. Torriani, Rob Knight, Jack Gilbert, Daniel A. Sweeney, Sarah M. Allard

**Affiliations:** 1 Department of Pediatrics, School of Medicine, University of California San Diego, La Jolla, CA USA; 2 Scripps Institution of Oceanography, University of California San Diego, La Jolla, CA USA; 3 Center for Microbiome Innovation, Jacobs School of Engineering, University of California San Diego, La Jolla, CA USA; 4 Department of Computer Science and Engineering, Jacobs School of Engineering, University of California San Diego, La Jolla, CA USA; 5 Division of Biostatistics, University of California, San Diego, La Jolla, CA USA; 6 Bioinformatics and Systems Biology Program, Jacobs School of Engineering, University of California San Diego, La Jolla, CA USA; 7 Microbiome Core, School of Medicine, University of California San Diego, La Jolla, CA USA; 8 Infection Prevention and Clinical Epidemiology Unit at UC San Diego Health, Division of Infectious Diseases and Global Public Health, Department of Medicine, UC San Diego, San Diego, CA USA; 9 IBM, T.J Watson Research Center, Yorktown Heights, New York, USA; 10 AI and Cognitive Software, IBM Research-Almaden, San Jose, CA USA; 11 IBM Research UK, The Hartree Centre, Warrington, UK; 12 Department of Bioengineering, University of California San Diego, La Jolla, CA USA; 13 Division of Pulmonary, Critical Care and Sleep Medicine, Department of Internal Medicine, University of California San Diego, La Jolla, CA USA

**Keywords:** Built environment, SARS-CoV-2, 16S rRNA, Microbiome, COVID-19

## Abstract

**Background:**

SARS-CoV-2 is an RNA virus responsible for the coronavirus disease 2019 (COVID-19) pandemic. Viruses exist in complex microbial environments, and recent studies have revealed both synergistic and antagonistic effects of specific bacterial taxa on viral prevalence and infectivity. We set out to test whether specific bacterial communities predict SARS-CoV-2 occurrence in a hospital setting.

**Methods:**

We collected 972 samples from hospitalized patients with COVID-19, their health care providers, and hospital surfaces before, during, and after admission. We screened for SARS-CoV-2 using RT-qPCR, characterized microbial communities using 16S rRNA gene amplicon sequencing, and used these bacterial profiles to classify SARS-CoV-2 RNA detection with a random forest model.

**Results:**

Sixteen percent of surfaces from COVID-19 patient rooms had detectable SARS-CoV-2 RNA, although infectivity was not assessed. The highest prevalence was in floor samples next to patient beds (39%) and directly outside their rooms (29%). Although bed rail samples more closely resembled the patient microbiome compared to floor samples, SARS-CoV-2 RNA was detected less often in bed rail samples (11%). SARS-CoV-2 positive samples had higher bacterial phylogenetic diversity in both human and surface samples and higher biomass in floor samples. 16S microbial community profiles enabled high classifier accuracy for SARS-CoV-2 status in not only nares, but also forehead, stool, and floor samples. Across these distinct microbial profiles, a single amplicon sequence variant from the genus *Rothia* strongly predicted SARS-CoV-2 presence across sample types, with greater prevalence in positive surface and human samples, even when compared to samples from patients in other intensive care units prior to the COVID-19 pandemic.

**Conclusions:**

These results contextualize the vast diversity of microbial niches where SARS-CoV-2 RNA is detected and identify specific bacterial taxa that associate with the viral RNA prevalence both in the host and hospital environment.

**Video Abstract**

**Supplementary Information:**

The online version contains supplementary material available at 10.1186/s40168-021-01083-0.

## Background

Severe acute respiratory syndrome coronavirus 2 (SARS-CoV-2) is the causative agent of a novel infectious disease, COVID-19, that has reached pandemic proportions. This pandemic has been characterized by sustained human to human transmission and has caused more than 91 million cases and nearly 2 million deaths worldwide (as of 15 January 2020, WHO report).

Viruses exist in complex microbial environments, and specific virus-bacterium interactions have been increasingly documented in host-associated contexts. In the animal microbiome, the gastrointestinal tract contains the greatest number and density of bacteria, and many virus-bacterium interaction studies have therefore focused on enteric viruses. Gut bacteria have been shown to directly modulate enteric virus infectivity via improving thermostability [1], increasing environmental stability [2], and encouraging viral genetic diversity and fitness [3]. Virus-bacterium interactions have also been observed in upper-respiratory tract infections including influenza A [4, 5] and oral human papillomavirus infection [6]. Most recently, prevalent bacteria in the human microbiome have been demonstrated to alter the human glycocalyx, thereby modulating the ability of SARS-CoV-2 to bind host cells [7].

In addition to observed virus-bacterium interactions in the host, existing evidence suggests that bacteria in indoor spaces (the “built environment”) may also influence viral stability or virulence. The risk of contracting SARS-CoV-2 is higher indoors than outdoors, particularly in poorly ventilated areas [8], and the built environment has a distinct microbiome [9]. The built environment microbiome is usually dominated by human-associated microbes [10]. It is estimated that humans shed approximately 37 million bacterial genomes per hour into their built environments [11]. In a study following the building of a new hospital, it was discovered that indoor spaces were seeded with microbes from patients and health care workers [12]. Bacterial load was found to positively correlate with viral load across a variety of surface types and humidity conditions in the built environment [13]. Given the nature of known virus-bacterium interactions, we hypothesized that associations between specific bacteria and SARS-CoV-2 may also be detectable in the built environment.

Despite evidence that SARS-CoV-2 can persist on surfaces under controlled conditions for days [14], more recent studies have demonstrated that fomite transmission is relatively low-risk in real world conditions [15–17]. Nevertheless, SARS-CoV-2 RNA detection has been widely reported across hospital surfaces [18–20]. To test whether specific bacterial taxa in the host or built environment co-associate with SARS-CoV-2, we collected samples from hospital surfaces, patients, and health care workers in the intensive care unit (ICU) and medical-surgical floor during the onset of the COVID-19 outbreak, screened for viral RNA presence, and sequenced the bacterial community.

## Results

### SARS-CoV-2 RNA detection across surfaces and patient samples

**Fig. 1:**
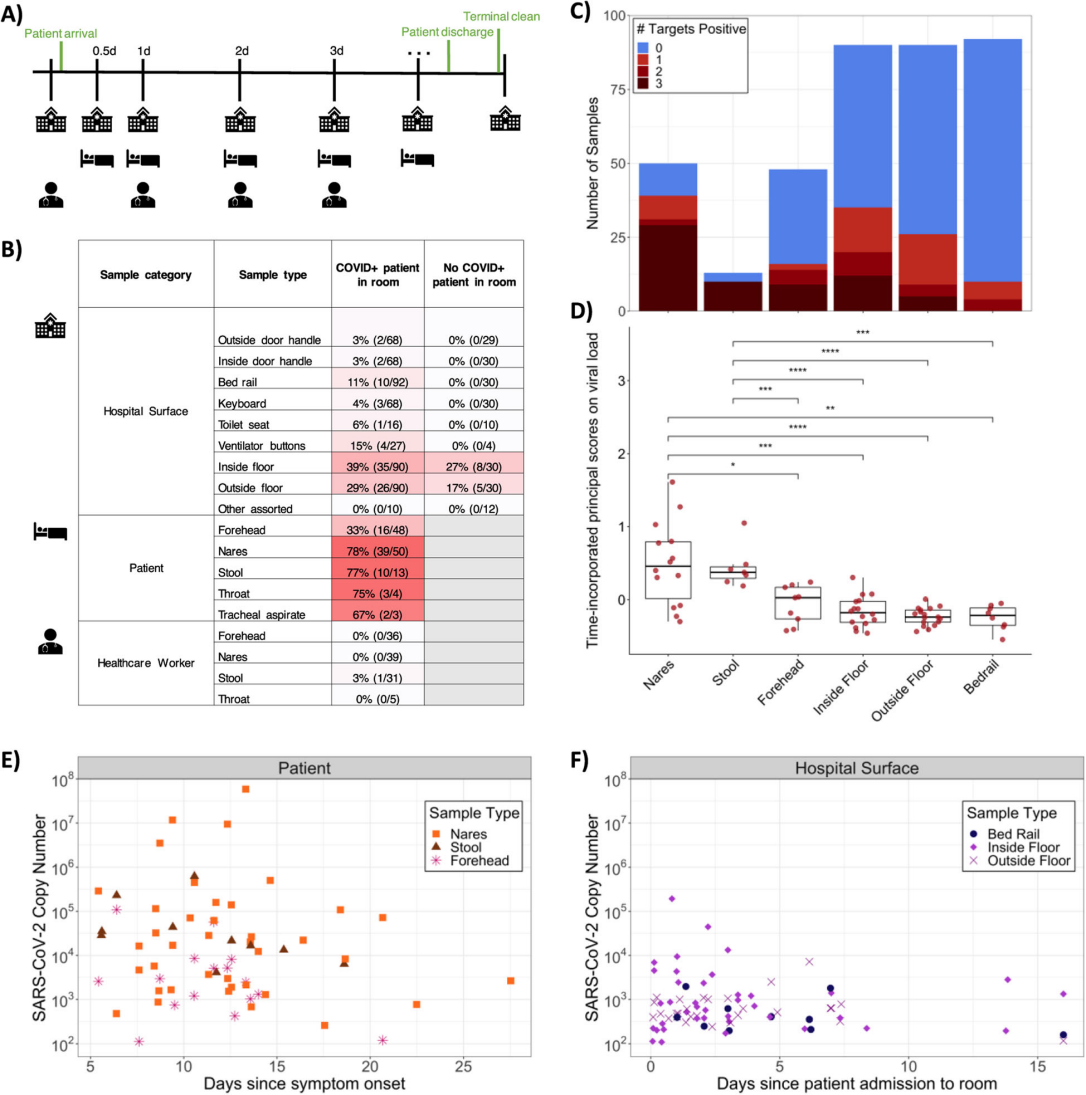
Summary of SARS-CoV-2 RNA detection in the dataset. **A** Schematic diagram of the experimental design highlighting the time frame for sample collection across sample types. **B** Percent and number of SARS-CoV-2 positives for each sample type collected from rooms occupied or not occupied by patients with COVID-19. Not occupied includes both post-cleaning rooms and rooms currently occupied by a patient negative for COVID-19. **C** Number of samples and SARS-CoV-2 screening results for 3 gene targets (N1, N2, and E-gene). **D** Boxplot of time-incorporated principal scores on viral copies per swab for different sample types. Each dot represents the functional principal component score for each viral load trajectory over time, which was estimated from sparse functional principal components analysis on viral load over time; *p < 0.05, **p < 0.01, ***p < 0.001, ****p < 0.0001, Wilcoxon signed-rank test. **E** Viral copies per swab relative to date of symptom onset across COVID-19 patient sample types, where only sample types with both n positive> 10 and % positive> 10% are included. **F** Viral copies per swab relative to date of room admission across hospital surface sample types, where samples from rooms occupied by a COVID-19 patient at the time of sampling are included. Again, sample types with both n > 10 and % positive> 10% are included

Sample collection for SARS-CoV-2 RNA screening is typically performed using viral transport media containing fetal bovine serum and a cocktail of antibiotics, which could negatively influence studies of bacteria and other microbes [21, 22]. For this study, swab samples were stored in 95% EtOH in order to inactivate the virus for safe transportation [23] while stabilizing the microbial community [24]. A total of 972 samples were collected longitudinally from 16 patients with clinical laboratory confirmed SARS-CoV-2 infection (118 samples), 10 health care workers assigned to these patients (113 samples), and 734 hospital surfaces either inside or immediately outside of the patients’ rooms over the span of two months (Fig. 1A). The 16 patients (5 females and 11 males) enrolled in this study ranged from age 20 to 84, with a median age of 49.5 years (Fig S1). Approximately 50% of patients were Hispanic/Latino, 31% were non-Hispanic/Latino White, 13% were Black, and 6% were Pacific Islander. Of the patients for whom antibiotic treatment information was collected, the majority had received at least one antibiotic. The number of days spent in the hospital ranged from 1 to 25, with a median stay of 9 days.

Each sample was screened for the presence of SARS-CoV-2 RNA using three distinct primer/probe sets: the U.S. Center for Disease Control N1 and N2 targets and the World Health Organization E-gene target (see methods). The US Food and Drug Administration has issued Emergency Authorization for more than 150 RT-qPCR assays for the detection of SARS-CoV-2, the majority of which define a positive result as amplification in a single target [25]. Accordingly, we designated samples as positive if at least one out of three targets amplified with a Ct value below 40.

Of the surfaces sampled, 13.1% contained detectable SARS-CoV-2 RNA, including those touched primarily by health care workers (keyboard, ventilator buttons, door handles inside, and outside the rooms) and those directly in contact with the patient (toilet seats and bed rails). A small number of other surface samples were collected (room air intake filter, n = 13; tap water, n = 4; health care worker shoes, n = 2; ultrasound buttons, n = 2; inside of veil box, n = 1), for which no SARS-CoV-2 RNA was detected (Fig. 1B). Of the patients enrolled in the study, we collected at least one positive sample from 15/16 patients (nares, forehead, or stool) and from 14/15 associated hospital rooms. In rooms where patient samples were not available, surfaces screened positive at least once for 6/6 COVID-19 patient rooms and 4/5 non-COVID-19 patient rooms. Floor samples had the highest positivity rates (36% of samples collected from the floor near the patients’ bed, i.e., “Inside Floor”, and 26% of samples collected from the floor immediately outside of the patient room, i.e., “Outside Floor”) (Fig. 1B, Fig. S2). In some cases, SARS-CoV-2 RNA was detected on the floors of rooms with non-COVID-19 patients and in rooms that had been cleaned following COVID-19 patient occupancy (Fig. 1B, Fig. S3C).

For the purposes of this study, viral load was defined as viral copies per swab extrapolated from Ct values of serially diluted viral RNA amplicons included on each plate (see methods). The surface area swabbed for built environment samples was consistent within sample types, and only three healthcare providers collected samples to reduce variation in swabbing technique. Most of the positive surface samples amplified only one or two out of the three SARS-CoV-2 targets (Fig. 1C) and had significantly lower viral load over time compared to patient nares and stool samples (*p* < 0.003, non-parametric test from sparse functional principal components analysis) [26], but similar viral load to patient forehead samples (Fig. 1D). SARS-CoV-2 viral load tended to decrease slightly in patients over time (Fig. 1E) but was detectable in patient’s nares up to 27 days after symptom onset. For a COVID-19-positive patient’s stay, viral load also tended to decrease slightly on associated hospital surfaces including bed rails and floor samples but remained detectable up to 16 days after patient admission (Fig. 1F). Due to high patient volume necessitating immediate room turnover, rooms were not left unoccupied long enough to collect repeated samples after patient discharge and room cleaning. The overall high Ct values on hospital surfaces suggest that the detected SARS-CoV-2 viral RNA was likely not in sufficient quantities to be infectious, consistent with previous findings of hospital surfaces [18, 19]. Of 113 health care worker samples, only one stool sample amplified for one of the three viral targets. No other samples collected from this health care worker, and no samples from any other health care worker treating patients with COVID-19 had any viral target amplification. Moreover, no health care workers in this study had detectable serum antibodies against SARS-CoV-2 during routine employee screening.

### Microbial context of SARS-CoV-2 RNA detection

**Fig. 2:**
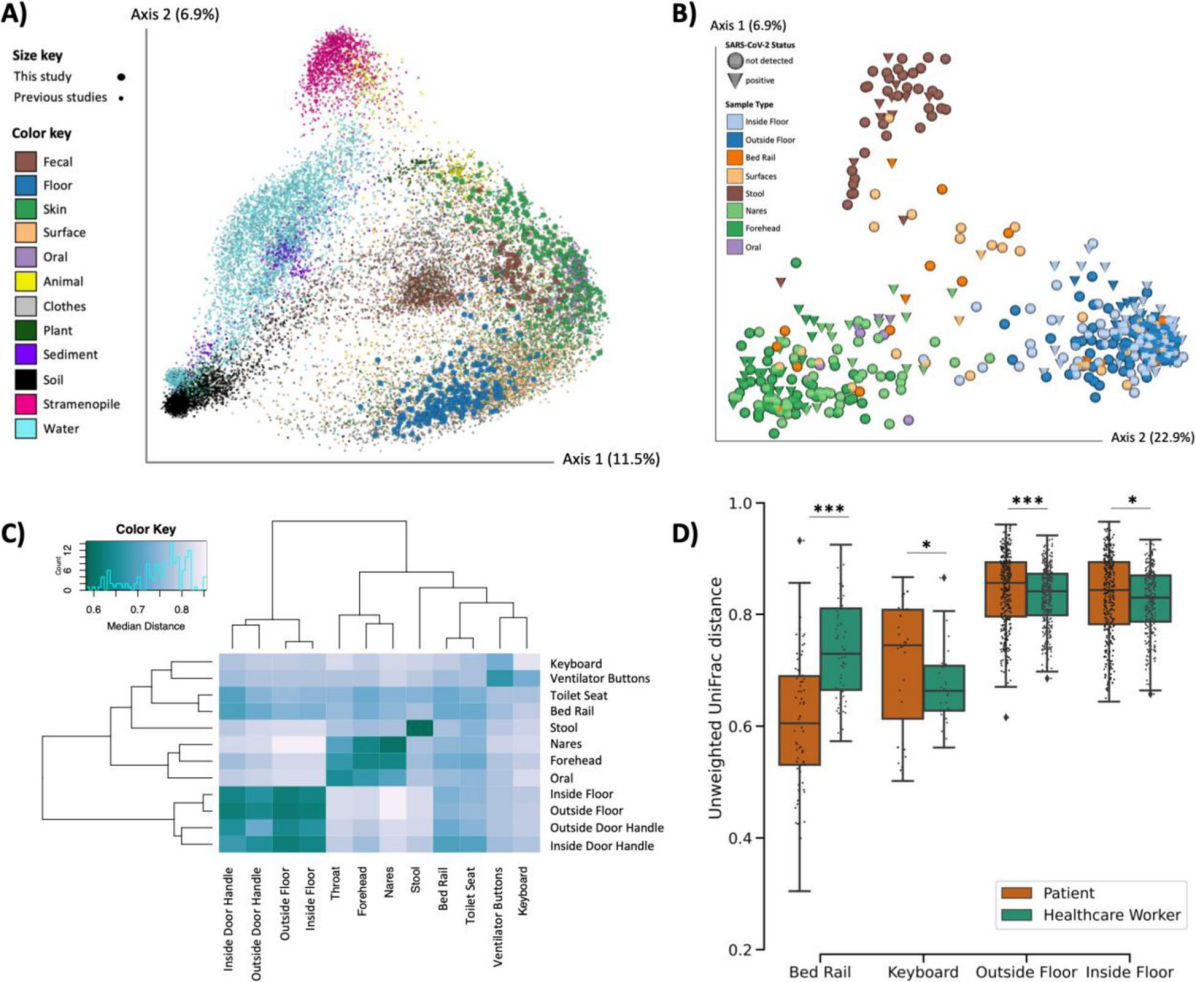
Microbial diversity of SARS-CoV-2 patients, health care workers, and the built environment in COVID-19 units. **A** Principal coordinates analysis (PCoA) of unweighted UniFrac distances comparing the Earth Microbiome Project meta-analysis (n = 19,497, small dots) and this study (n = 591, large dots). **B** PCoA of unweighted UniFrac distances in this study. **C** Heatmap of unweighted UniFrac distance among surface and patient sample types. Diagonal lines represent median distances within individual sample types. **D** Pairwise unweighted UniFrac distance between the human surface (i.e., forehead and nares) and their paired surface samples. Statistics represent bootstrapped Kruskal-Wallis; *p < 0.05, **p < 0.01, ***p < 0.001

To compare the built environment microbial communities in this study to that in prior studies, we performed 16S V4 rRNA gene amplicon (16S) sequencing on all samples including both positive and negative controls to exclude failed samples according to the KatharoSeq protocol (see methods) [27]. A total of 589 out of the 972 samples passed quality filtering. Most of the sample dropouts were low biomass samples from surfaces in the built environment (49% of hospital surface samples compared to 9% of human samples). Fewer samples that failed 16S sequencing were SARS-CoV-2 positive (6.7%) compared to samples that sequenced successfully (23.9%). A meta-analysis with samples from the Earth Microbiome Project [28], an intensive care unit microbiome project [29], and a hospital surface microbiome study performed at another hospital [12] (a total of 19,947 samples collected and processed using comparable and standardized Earth Microbiome Project methods [28, 30]) contextualized the microbial composition of samples from this hospital study and the microbial diversity covered in this dataset (Fig. 2A). Using source-tracking [31] on the meta-analysis dataset, we found that floor samples, which clustered separately from the rest of our dataset (Fig. 2C), were similar to built environment samples from previous studies (Fig. S4).

Beta diversity estimated using unweighted UniFrac distances [32] in this study showed that floor samples, stool samples, and nares/forehead samples formed three distinct clusters with other surfaces falling between the human skin and floor samples (Fig. 2B-C). SARS-CoV-2 viral load was weakly correlated with unweighted UniFrac beta diversity (PERMANOVA R^2^ < 0.01, p value = 0.043, Fig. S5).

We compared beta diversity between human samples and paired built environment samples from the patients’ respective hospital rooms. As expected, microbial composition of high-touch surfaces routinely used by healthcare workers, such as keyboards and floor samples, were significantly more similar to health care worker samples, whereas samples from bed rails that are frequently touched by patients were significantly more similar to the patient samples (Fig. 2D), reflecting likely inputs of microbes to these communities. Notably, the percent of SARS-CoV-2 positive bed rail samples was lower than floor (11% vs. 39%) despite the high similarity of bed rail microbiomes to the corresponding patient microbiomes.

### Microbial diversity and biomass positively associated with SARS-CoV-2

**Fig. 3:**
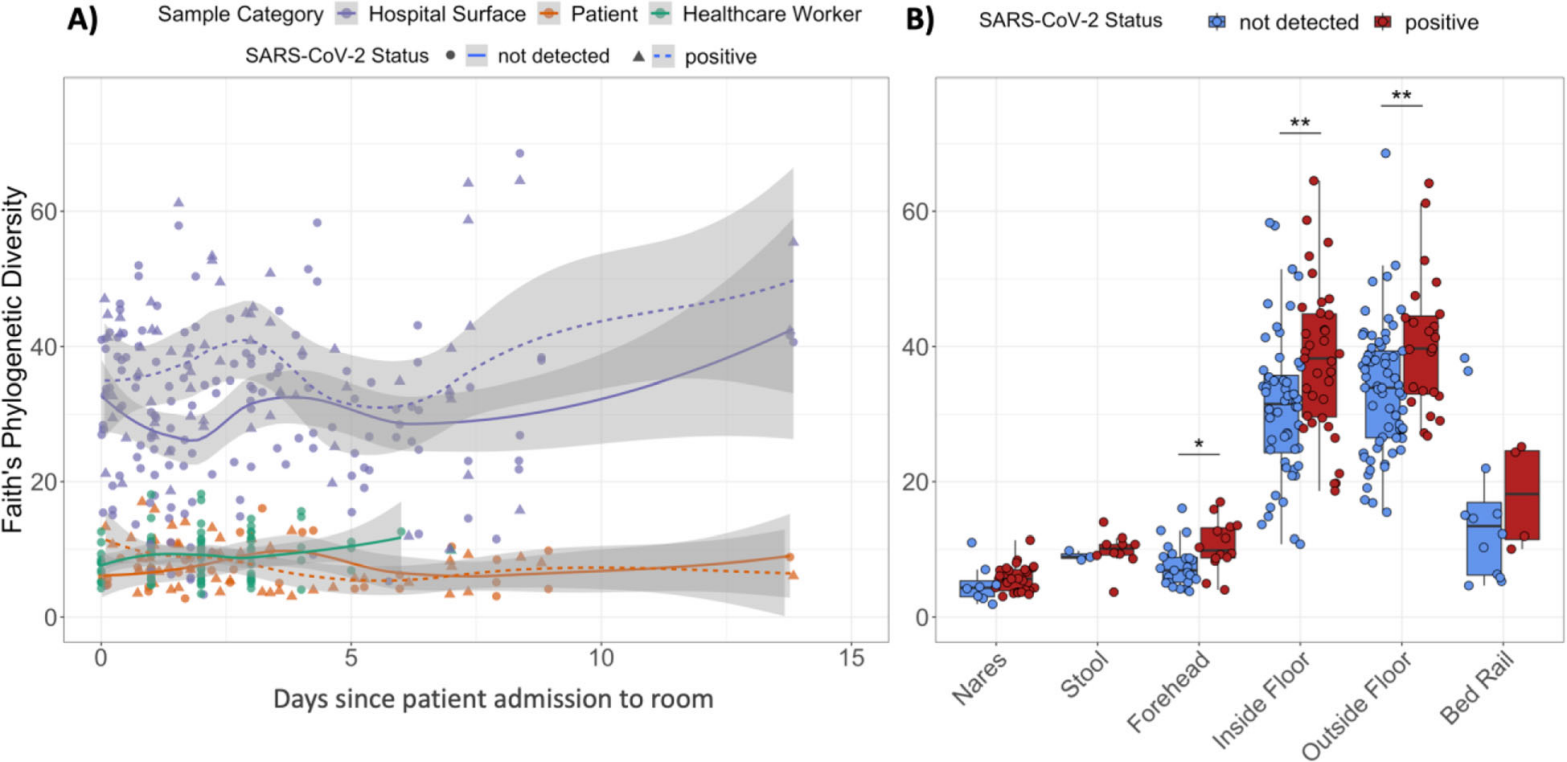
Alpha diversity is higher in SARS-CoV-2 positive samples of each type. **A** Faith’s phylogenetic diversity (rarefied to 4000 reads per sample) of human and surface samples over time, fitted with locally estimated scatterplot smoothing (LOESS) curves. **B** Faith’s phylogenetic diversity of humans and their surface samples grouped by SARS-CoV-2 screening results. Statistics resulted from Wilcoxon signed rank tests; *p < 0.05, **p < 0.01

Next, we tested whether bacterial alpha diversity is associated with SARS-CoV-2 RNA detection. Overall, Faith’s phylogenetic alpha diversity was significantly higher among surface samples than patient or health care worker samples (Fig. 3A). Faith’s phylogenetic diversity was significantly higher for SARS-CoV-2 positive samples in forehead, inside floor, and outside floor samples (Fig. 3B).

The high alpha diversity of floor samples and significant association with SARS-CoV-2 RNA detection led us to examine potential differences in biomass across floor samples. 16S read count and human RNAse P Ct values are indirect measures of total bacterial and human biomass, respectively, and were significantly correlated (Pearson R^2^ = − 0.40, p < 0.0001). 16S read count was significantly higher in floor samples with detected SARS-CoV-2 RNA, but did not correlate with the number of viral copies detected per swab (Fig. S6A). The abundance of human RNAse P was also significantly higher in floor samples with SARS-CoV-2 RNA, and positively correlated with viral load (Pearson R^2^ = − 0.31, p value = 0.011) (Fig. S6B); this correlation was not observed for the other sample types examined (nares, forehead, stool, bed rail). These results suggest that increased detection of SARS-CoV-2 RNA on floors could be related to the relatively high load of total microbial and human biomass compared to other surfaces.

To determine the relationship between abundance of SARS-CoV-2 RNA and bacterial composition in the built environment, we performed forward stepwise redundancy analysis [33] on unweighted UniFrac [34] principal components from floor samples (n = 215). We chose floor samples for this analysis since floor samples had the largest number and highest biomass of all surfaces sampled (Fig. S7). Three non-redundant variables had a significant effect size, explaining a total of 21.7% variation in the data (Fig. S6C). The variable with the strongest effect size was patient identity (17.5%, p value = 0.0002), which aligns with previous work demonstrating that the built environment microbiome is contributed from the humans inhabiting that space [12]. Whether the sample was an inside floor sample (next to patient bed) or outside floor sample (hallway directly in front of patient room) also had a small, yet significant effect size (0.8%, p value = 0.04). Importantly, SARS-CoV-2 detection status also significantly contributed to microbial variation (3.4%, p value = 0.0004).

### Unique microbial signatures predict SARS-CoV-2 across patient sample types

**Fig. 4:**
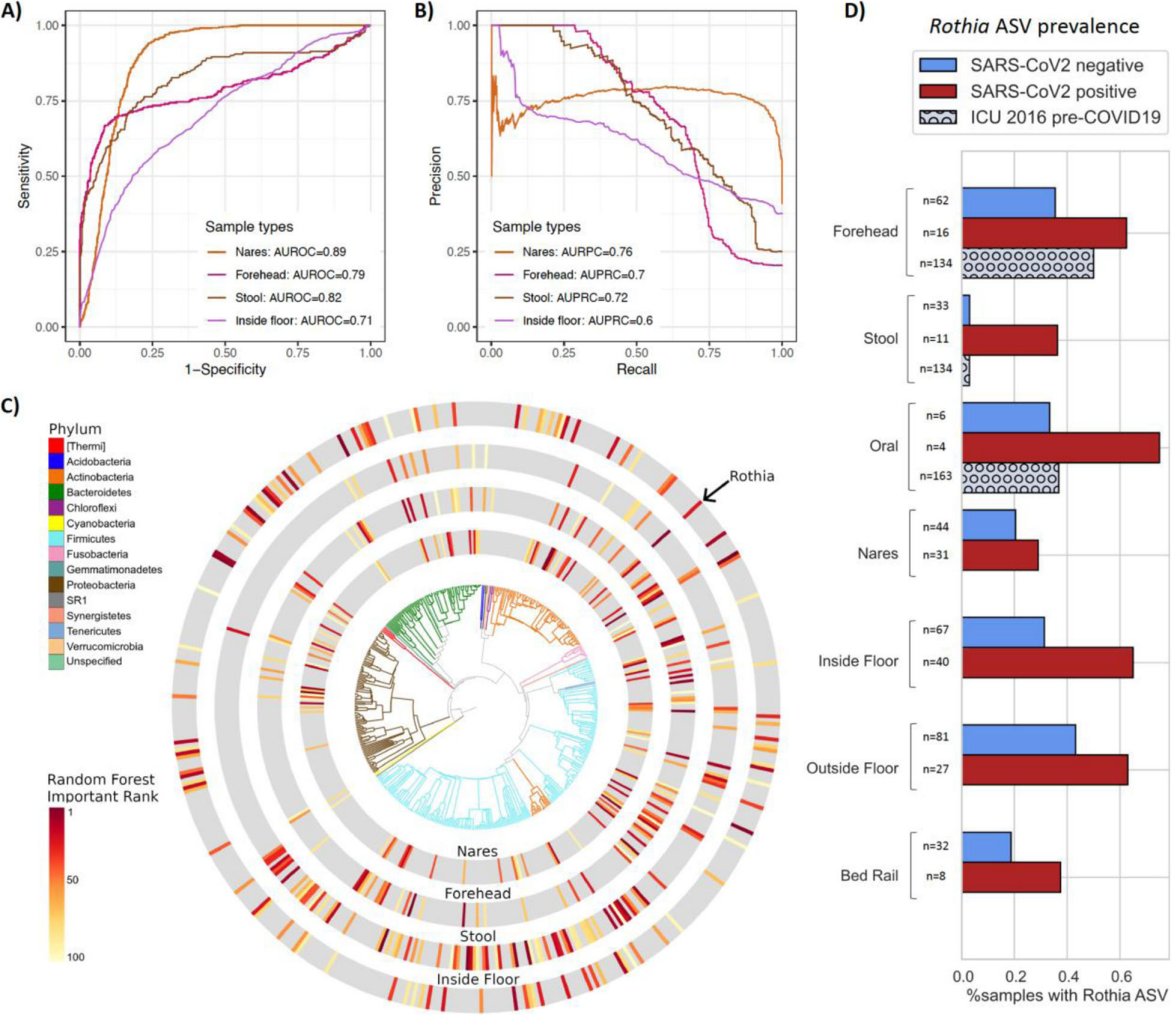
Bacterial composition is predictive of SARS-CoV-2 status in nares, forehead, stool, and inside floor samples. The prediction performance of random forest classifiers on SARS-CoV-2 status for each sample type was assessed using AUROC (**A**) and AUPRC (**B**) for nares (n = 76), forehead (n = 79), stool (n = 44), and inside floor (n = 107), in a 100-fold cross-validation approach (see methods). **C** EMPress plot of the 100 features most predictive of SARS-CoV-2 status in nares, forehead, stool, and inside floor samples, where a single ASV with 100% alignment to *Rothia dentocariosa* was identified across all sample types. **D** Proportion of samples containing the highly predictive *Rothia dentocariosa* ASV in SARS-CoV-2 positive and negative samples from the current study and from [29] (ICU 2016 pre-COVID19)

To identify microbial features associated with SARS-CoV-2 positive samples, we independently trained random forest (RF) classifiers on nares (N = 76), stool (N = 44), and forehead samples (n = 79) from patients with COVID-19 and health care workers. Based on 16S rRNA gene amplicon sequencing microbial profiles, the RF models predicted SARS-CoV-2 status (positive vs. not detected) with 0.89 area under the receiver operating characteristic curve (AUROC) in unseen nares samples (Fig. 4A). Strikingly, skin (AUROC = 0.79) and stool (AUROC = 0.82) also showed high classifier accuracy. As the SARS-CoV-2-negative samples were overrepresented in the data, we also employed the area under the precision recall curves (AUPRC) to evaluate the prediction performance of each classifier, which were 0.76, 0.72, and 0.7 for nares, stool, and forehead, respectively (Fig. 4B). A RF model built from bacterial profiles on the inside floor also showed a moderate prediction accuracy for discriminating SARS-CoV-2 status (AUROC = 0.71; AUPRC = 0.6, Fig. 4A and B). RF classifiers trained on outside floor and bed rail samples did not perform well, especially in the precision-recall curves (Fig. S8).

The phylogenetic relationship of the top 100 ranked amplicon sequence variants (ASV) from the RF models were visualized with EMPress [35] (Fig. 4C). Stool and inside floor samples each had distinct sets of taxa driving the RF model compared to nares and forehead samples, which were more similar to one another. Many of the highly ranked ASVs in the stool samples are from the class *Clostridiales*, a polyphyletic group of obligate anaerobes that were also identified as predictive of SARS-CoV-2 status in a wastewater study [36].

ASVs from the genera *Actinomyces*, *Anaerococcus*, *Dialister*, *Gemella*, and *Schaalia* were in the top 40 ranked predictive features of both forehead and nares samples (Table S2); these taxa are normally found in anterior nares samples [37–39], but are not commonly described in forehead microbiome samples. Interestingly, from Fig. 2C, we observed that the unweighted UniFrac distance between samples from the same individual’s nares and forehead were more similar in rooms with SARS-CoV-2 positive surfaces, suggesting that patients who shed virus into their environment could be cross-contaminating bacteria between nares and forehead (Fig. S9).

One ASV with an exact match to *Rothia dentocariosa* (GenBank ID CP054018.1) was highly ranked as predictive across all four disparate sample types: nares, forehead, stool, and inside floor (Fig. 4C). There were a total of 36 ASVs aligning to the genus *Rothia*, of which only 7 ASVs were present in > 2% of samples. Of these, the only ASV found to associate with viral presence was the *Rothia* ASV presented here. Further investigation shows this ASV is more prevalent in SARS-CoV-2 positive samples across all sample types examined. To exclude the possibility that this *Rothia* ASV was associated with sick patients generally, we examined the prevalence of this ASV in an intensive care unit microbiome study that was performed in 2016 [29] and found that high prevalence of this *Rothia* ASV is specific to SARS-CoV-2 positive patient samples (Fig. 4D). We also found that patients with cardiovascular disease comorbidities tended to have higher prevalence of the *Rothia* ASV associated with SARS-CoV-2, compared to patients without pre-existing cardiovascular disease (45% versus 26%, respectively). *Rothia dentocariosa* can cause endocarditis, particularly in patients with a history of cardiovascular disease [40, 41]. Using data from the American Gut Project [42], we tested for the presence of this *Rothia* ASV in samples from those self-reporting a medical diagnosis of a cardiovascular disease and those self-reporting not having a cardiovascular disease. We observed a significantly higher prevalence of the *Rothia* ASV in samples with a reporting of cardiovascular disease (Fisher’s exact test, p = 0.041) than those without, suggesting that the *Rothia* ASV could be associated with cardiovascular disease outside of the context of SARS-CoV-2.

## Discussion

The COVID-19 pandemic continues unabated as outbreaks ebb and flow around the globe. Because evidence for the synergistic effects of host-associated bacteria on multiple viral pathogens continues to emerge, we set out to identify possible correlations between host- or surface-associated bacteria and SARS-CoV-2 RNA presence and abundance in the hospital built environment. At the onset of sampling, no hospital rooms or health care workers enrolled in the study had known exposure to SARS-CoV-2. Although both patient samples and surface samples from patient rooms tested positive throughout the study, all samples collected from health care workers providing direct patient care to patients with COVID-19 were negative by both clinical RT-qPCR and antibody tests (data not shown). This includes the 3 health care workers who collected samples for the study. Aside from one stool sample where one of three viral targets amplified in our screening, all health care worker samples in this study (n = 113) were negative for SARS-CoV-2, similar to findings from previous studies of exposed health care workers using airborne, contact, and droplet protective PPE [43–45]. This contrasts with early reports of high SARS-CoV-2 transmission levels among health care workers before the implementation of general hospital-wide masking of healthcare workers and patients and of eye protection when interacting with unmasked patients [46, 47]. Our findings are thus consistent with other work directly testing the importance of providing healthcare workers with appropriate PPE and rigorous training in donning and doffing procedures to minimize self-contamination.

The demographics of participants in this study reflected global trends demonstrating that race and ethnicity, as well as sex, influence susceptibility to SARS-CoV-2 as well as clinical outcomes. The majority of subjects enrolled were men, who are generally more at risk for worse outcomes when infected by COVID-19 [48]. Compared with the demographics of San Diego, the distribution of patient ethnicities is in line with the CDC reporting that Black and Hispanic people are more likely to be hospitalized with COVID-19 compared to non-Hispanic White people [49]. Due to sample size constraints, analyses of the influence of these demographics on SARS-CoV-2 prevalence and microbial associations with SARS-CoV-2 were not possible.

In this study, approximately 16% (83/529) of surface samples from hospital rooms occupied by patients with COVID-19 and 6% (13/205) of surface samples from hospital rooms not currently occupied by patients with COVID-19 had detectable levels of SARS-CoV-2 RNA. Of the various surfaces sampled in this study, floor samples had the highest prevalence of SARS-CoV-2 RNA detection. The intense and frequent oropharyngeal, respiratory, skin, and bowel care provided to these critically ill patients is expected to produce shedding and contamination of the environment in close proximity to the patient, including the floors. Our findings replicate previous studies where floors had the highest prevalence of SARS-CoV-2 RNA of all hospital room surfaces tested [20, 50]. Previous studies of environmental contamination reported higher surface prevalence of SARS-CoV-2 in hospital settings, ranging from 25% to over 50% [18, 50–52]. The lower SARS-CoV-2 prevalence rates in this study could be due to differences in sampling strategy (e.g., area sampled, storage and extraction methods), more careful environmental cleaning of high touch areas around the patient, or due to physiological differences since different surface types differentially influence viral RNA persistence [53]. Furthermore, contamination of hospital room surfaces with SARS-CoV-2 tends to be highest during the first 5 days after symptom onset [50]. All patients enrolled in our study had symptoms for at least 6 days before admission to the hospital and enrollment in this study.

While SARS-CoV-2 RNA was identified via RT-qPCR for both patient and hospital room samples, it is important to note that this study did not determine whether the detected viral RNA was viable or infectious. Two studies assaying infectivity of surface and air samples using RT-qPCR in parallel showed that samples with Ct values over 30 were not infectious [18, 19]. In our study, only 2 out of 79 positive surface samples amplified at least one SARS-CoV-2 target under 30 cycles. Both of these samples were from the floor directly next to the patient bed in rooms that hosted patients who were mechanically ventilated during their stay.

It should be acknowledged that transportation of samples in ethanol (to ensure the safety of those handling samples, as well as to enable microbiome analysis) instead of using viral transport media may have resulted in overall lower viral RNA yield. Despite these potential sources of variation, we found that the microbiomes found on bed rails and corresponding patient microbiomes were highly similar to one another before cleaning, but this similarity disappeared after environmental cleaning was performed. Microbial community composition was also more similar between humans and the surfaces they touched (including between health care workers and keyboards, as well as patients and bed rails), supporting the robustness of our microbial sample collection and processing protocols.

It is both a strength and a limitation of this study that standard of care environmental cleaning was performed and was not influenced or altered by the study team. The daily cleaning regimen can vary depending on staff and other factors (hospital room surface types and disinfection protocols are summarized in Table S1) which is representative of hospital environmental practices worldwide. To limit additional burden on hospital staff, specific cleaning events were not tracked, except for cleaning after patient discharge. SARS-CoV-2 RNA was amplified from floor samples, albeit at a relatively low abundance based on Ct values, even in rooms with non-COVID-19 patients and after patient discharge cleaning. Although transmission risk from the floor is likely negligible as discussed above, resuspension of particles from the floor in highly transited areas cannot be ruled out. In this study, the relatively high positivity rate for floor samples allowed us to use them as a proxy to study how microbial communities are interrelated with shed virus.

In the built environment, microbial load, human biomass, and alpha diversity were higher in floor samples positive for SARS-CoV-2. More controlled sampling procedures are required to determine if the increased alpha diversity associated with SARS-CoV-2 positive samples is due to increased biomass or if it is more specifically correlated with SARS-CoV-2 RNA presence. Floor samples had the highest biomass of all the surface samples tested, including high-touch surfaces (e.g., bed rail, keyboard, door handles). This may help explain the higher prevalence of positive floor samples in COVID-19 patient rooms (39%) versus bed rail samples (11%), despite their distance from the patient. This is in agreement with previous research showing that bacterial and viral load are positively correlated in built environment samples [13]. The relatively low prevalence of SARS-CoV-2 contamination on bed rail samples may also be because many of the patients were deeply sedated and were not actively moving in bed, including touching the bed rails, or because high touch areas in close proximity to the patient are cleaned by nurses at each shift, and/or due to differences in material (vinyl versus plastic).

Using random forest models to classify microbes associated with SARS-CoV-2 RNA detection, we found 16S microbial profiles had high predictive accuracy of SARS-CoV-2 RNA presence in nares, stool, forehead, and inside floor samples. Despite these sample types having distinct microbiomes covering a broad range of microbial diversity (Fig. 2), we identified a single *Rothia* ASV that was highly ranked in the random forest classifier across all four sample types. This ASV was also more prevalent in SARS-CoV-2 positive samples across all human sample types and floor and bed rail samples in our dataset. By comparing the prevalence of this ASV across our dataset and a 2016 study from an intensive care unit [29], we found that this signal is specific to SARS-CoV-2 positive samples, and not other factors associated with an ICU admission such as antibiotic use. This finding supports previous work reporting *Rothia* to be enriched in SARS-CoV-2 positive stool [54] and bronchoalveolar lavage fluid [55] and further suggests a role in nares, forehead, and surfaces. These results further suggest that there may be species- or strain-level specificity to these dynamics.

While the mechanism remains unclear, the consistent *Rothia* ASV prevalence trend across both patient and surface sample types suggests an association of this bacteria with SARS-CoV-2. Although this study was carried out at a single hospital, and built environment microbiomes tend to vary based on location and occupancy [56], previous research into the clinical relevance of *Rothia* species indicates that this association warrants further investigation. Species from the genus *Rothia* are common to the human oral microbiome [57], but have also been identified as opportunistic pathogens [40]. Oral microbes have been found to colonize the gastrointestinal tract, especially in disease states [58]. This, along with our finding of the predictive nature of the *Rothia* ASV in stool, may suggest a possible increased oral-fecal transmission triggered under viral infection that manifests as a hallmark of COVID-19. Furthermore, the specific *Rothia* ASV identified in this study appears to associate with cardiovascular disease even in people without SARS-CoV-2 infection, indicating that *Rothia* may be a marker for individuals at increased risk from COVID-19. Cardiovascular disease can predispose individuals to worse outcomes with COVID-19, and SARS-CoV-2 infection has been associated with cardiovascular complications [59]. Further studies are required to determine the mechanism underlying the association between *Rothia* and SARS-CoV-2, the role of co-morbidities, and how this knowledge may be translated into effective methods for reducing SARS-CoV-2 virulence.

To better understand how virus-bacteria interactions influence pathogen infection, transmission, and health outcomes, studies using animal models could be useful and ultimately lead to the development of effective clinical interventions. In built environments, the findings from our study highlight the need to better understand viral distribution patterns and how bacterial distribution and abundance influence the persistence and viability of viruses, especially in the context of human health. Hospitals are promising study sites for these investigations, as they contain patients harboring known diseases, environmental factors are kept fairly consistent and regularly monitored, and standard of care consistency across facilities may allow for some extrapolation beyond each specific building investigated. These future studies could illuminate the development of viral pathogen mitigation strategies in both patients and the built environment.

## Conclusions

This large-scale study is the first to examine the microbial context of SARS-CoV-2 in a hospital setting. We detected SARS-CoV-2 RNA contamination across a variety of surfaces in the ICU and the general medical-surgical unit, including rooms that were not currently used to treat patients with COVID-19 infection. RT-qPCR results are not indicative of infectious virus; nevertheless, we were able to identify bacterial signatures predictive of SARS-CoV-2 RNA detection using a random forest model. Across a remarkable diversity of microbiomes (floor, nares, stool, skin), we identified a single bacterial ASV, *Rothia dentocariosa*, that was highly predictive of and co-identified with SARS-CoV-2 RNA. Our discovery of bacterial associations with SARS-CoV-2 both in humans and the built environment suggests that bacteria-virus synergy likely plays a role in the COVID-19 pandemic.

## Materials and methods

### Study design

Patients admitted to the UCSD Medical Center - Hillcrest who were either confirmed patients with COVID-19 or Persons Under Investigation (PUI: have symptoms and undergoing testing) were approached for informed consent upon admission. Patients whose clinical test was negative were included in the study as controls for surface sampling. Health care workers providing direct care for PUIs and patients with COVID-19 were included in the study. Following hospital policy, all underwent daily symptomatic screening and wore the following PPE during treatment of PUI and patients with COVID-19: goggles or face-shield, N95 mask, gown, gloves; hair and shoe coverings were not part of the required PPE but were available and inconsistently used. All participants were consented under UCSD Human Research Protections Program protocol 200613.

We followed the excretion pattern of the virus from the skin, respiratory tract, and gastrointestinal tract. From patients and health care workers, specimen samples were obtained from the forehead, nares, and stool. Additional throat swabs and/or tracheal aspirate samples were collected for a subset of patients and health care workers: “oral” samples. Patient samples were collected by gloved health care workers via dual-tipped synthetic swabs (BD BBL CultureSwabs #220145) which were immediately transferred to tubes containing 95% ethanol. Stool was collected from patient bed pans or from collection bags that were connected to a rectal tube. Health care workers self-collected swabs over a time series of 4 days. A chronological series was also employed for patient samples, with the target sampling schemes as follows: samples collected within the first 12 h of hospital admission with sequential samples obtained once daily for the first 4 days of hospitalization and a subset of samples collected regularly until the patient vacated the room (Fig. 1A). Actual sample collection timing varied by patient availability and duration in the hospital (Fig. S3).

Dual-tipped synthetic swabs (BD BBL CultureSwabs #220145) were pre-moistened by dipping for 5 seconds into 95% spectrophotometric-grade ethanol solution (Sigma-Aldrich #493511), and then used to vigorously swab surfaces that are frequently in contact with health care workers or patients. Surfaces were swabbed for 10–15 s with moderate pressure on a defined surface area, and swabs were returned to the collection container. Outside of patient rooms, prior to entering the room, the floor (1 square foot outside the entrance) and outside door handle were swabbed. Inside patient rooms, the inside door handle, floor (1 square foot near the patient’s bed on side closest to door), bedrail (side closest to door), and keyboard were swabbed. Depending on the patient room, if an air filter was present, the intake was swabbed. For a subset of samples, patient care equipment such as portable ultrasound and ventilator screen were also swabbed, as well as the toilet seat. After sample collection, dual-tipped swabs were returned to the swab container. Surface samples were collected at the same time as patient sample collection, as well as prior to patient admission and following patient discharge and room cleaning, when possible.

### Nucleic acid extraction

Sample plating and extractions of all clinical and environmental specimens were carried out in a biosafety cabinet Class II in a BSL2+ facility. Sample swabs were plated into a bead plate from the 96 MagMAX™ Microbiome Ultra Nucleic Acid Isolation Kit (A42357 Thermo Fisher Scientific, USA). Following the KatharoSeq low biomass protocol [27], each sample processing plate included eight positive controls consisting of 10-fold serial dilutions of the ZymoBIOMICS™ Microbial Community Standard (D6300 Zymo, USA) ranging from 5 to 50 million cells per extraction. Each plate also contained a minimum of 8 negative controls (sample-free lysis buffer). Nucleic acids purification was performed on the KingFisher Flex^TM^ robots (Thermo Fisher Scientific, USA) using the MagMAX^TM^ Microbiome Ultra Nucleic Acid Isolation Kit (Applied Biosystems^TM^), as instructed by the manufacturer. Briefly, 800 μL of lysis buffer was added to each well on the sample processing plate and briefly centrifuged to bring all beads to the bottom of the plate. Sample swab heads were added to the lysis buffer and firmly sealed first with MicroAmp™ clear adhesive film (Thermo Fisher Scientific, UK) using a seal roller, and the sealing process repeated twice using foil seals. The plate was beaten in a TissueLyser II (Qiagen, Germany) at 30 Hz for 2 min and subsequently centrifuged at 3700×*g* for 5 min. Lysates (450 μL/well) were transferred into a Deep Well Plate (96 well, Thermo Fisher Scientific, USA) containing 520 μL of MagMax^TM^ binding bead solution and transferred to the KingFisher Flex^TM^ for nucleic acid purification using the MagMax^TM^ protocol. Nucleic acids were eluted in 100 μL nuclease free water and used for downstream SARS-CoV-2 real time RT-qPCR.

### SARS-CoV-2 RT-qPCR and viral load quantification

The Center for Disease Control (CDC) 2019-Novel Coronavirus Real-Time RT-PCR Diagnostic Panel [60] and the E-gene primer/probe from the World Health Organization [61] were used to assess SARS-CoV-2 status via reverse transcription, quantitative polymerase chain reaction (RT-qPCR). Accordingly, each plate of extracted nucleic acid (96-well plate) was aliquoted into a 384-well plate with four separate reactions per sample; two reactions targeted the SARS-CoV-2 nucleocapsid gene (CDC N1 and N2), one reaction targeted the SARS-CoV-2 virporin forming E-gene (WHO E-gene), and one reaction targeted the human RNAse P gene as a positive control for sample collection and nucleic acid extraction (CDC RP).

Each reaction contained 3 μL of TaqPath^TM^ 1-Step RT-qPCR Master Mix (Thermo Fisher Scientific, USA), 400 nm forward and reverse primers and 200 nm FAM-probes (IDT, USA—table with sequences below), 4 μL RNA template, and H_2_O to a final volume of 10 μL. Master mix and sample plating were performed using an EpMotion automated liquid handler (Eppendorf, Germany). Each plate contained both positive and negative controls. The positive control was vRNA and eight serial dilutions of viral amplicons for viral load quantification (details below). Six extraction blanks and one RT-qPCR blank (nuclease-free H_2_O) were included per plate as negative controls. RT-qPCR was performed on the CFX384 Real-Time System (BIO-RAD). Cycling conditions were reverse transcription at 50 °C for 15 min, enzyme activation at 95 °C for 2 min, followed by 45 cycles of PCR amplification (denaturing at 95 °C for 10 s; annealing/extending at 55 °C for 30 s). Cycle threshold (Ct) values were generated using the CFX384 Real-Time System (BIO-RAD) software.

Viral load quantification was performed using a standard ladder comprising serially diluted target amplicons which was included in the RT-qPCR of each extraction plate, in place of the KatharoSeq control samples. SARS-CoV-2 viral RNA was reverse transcribed into cDNA using the Superscript IV enzyme (Thermo Fisher, USA) and PCR amplified with KAPA SYBR® FAST qPCR Master Mix (KAPA Biosystems, USA) using the N1, N2, and E gene primers in duplicate 20 μL reactions with cycling parameters as detailed above. Each amplicon reaction was run across a 1.5% agarose gel and the resulting bands were excised and purified into 100 μL nuclease-free water with the MinElute Gel Extraction Kit (Qiagen, Germany). Amplicons were quantified with in duplicate with the Qubit™ dsDNA HS Assay Kit (Thermo Fisher, USA) and copies per μL were calculated based on predicted amplicon length (N1 72 bp, N2 67 bp, and E gene 113 bp). Eight, 10-fold serial dilutions were added to the RT-qPCR for final estimated copy input per reaction ranging from 10 million to one. The limit of detection was between 10 and 100 vRNA copies per reaction, and the Ct values were highly consistent across extraction plates. Viral load per swab head was calculated by first using the slope and intercept from the N1 amplicon ladder linear regression per plate to determine the number of viral copies per reaction, and then multiplying this number by 25 since 4 μL out of a total 100 μL extracted nucleic acid was used as input to the RT-qPCR.

**Table:** 

Primer/probe	Sequence (5′ -> 3′)
2019-nCoV_N1-F	GAC CCC AAA ATC AGC GAA AT
2019-nCoV_N1-R	TCT GGT TAC TGC CAG TTG AAT CTG
2019-nCoV_N1-P	FAM-ACC CCG CAT TAC GTT TGG TGG ACC-BHQ1
2019-nCoV_N2-F	TTA CAA ACA TTG GCC GCA AA
2019-nCoV_N2-R	GCG CGA CAT TCC GAA GAA
2019-nCoV_N2-P	FAM-ACA ATT TGC CCC CAG CGC TTC AG-BHQ1
RP_F	AGA TTT GGA CCT GCG AGC G
RP_R	GAG CGG CTG TCT CCA CAA GT
RP_P	FAM – TTC TGA CCT GAA GGC TCT GCG CG – BHQ-1
E_Sarbeco_F1	ACAGGTACGTTAATAGTTAATAGCGT
E_Sarbeco_R2	ATATTGCAGCAGTACGCACACA
E_Sarbeco_P1	56-FAM/AC ACT AAG C/ZEN/C ATC CTT ACT GCG CTT CG/3IABkFQ/

### 16S rRNA gene amplicon sequencing

16S rRNA gene amplification was performed according to the Earth Microbiome Project protocol [28]. Briefly, Illumina primers with unique reverse primer barcodes [62] were used to amplify the V4 region of the 16S rRNA gene (515f-806rB, [63]). Amplification was performed in a miniaturized volume [64], with single reactions per sample [65]. Equal volumes of each amplicon were pooled, and the library was sequenced on the Illumina MiSeq sequencing platform with a MiSeq Reagent Kit v2 and paired-end 150 bp cycles.

### Statistical analysis

#### Data pre-processing

Raw 16S rRNA gene amplicon sequencing data was demultiplexed, quality filtered, and denoised with deblur [66] through Qiita [67] under study ID 13092. Downstream data processing was performed using Qiime2 [68]. Eight negative controls (blanks) and eight positive controls (serially diluted mock communities) were included in each 96-well extraction plate (see the “Nucleic acid extraction*”* section). The serially diluted mock communities included in each extraction plate were used to identify the read count threshold at which 80% of sequencing reads aligned to the positive control according to the KatharoSeq protocol [27] (code available at https://github.com/lisa55asil/KatharoSeq_ipynb), and all samples falling below the threshold set for each independent sequencing run were removed from downstream analysis. The KatharoSeq-filtered feature tables were merged, and features present in less than three samples were removed from downstream analysis, with the final feature table containing 589 samples and 9461 features.

#### Beta diversity analyses

To verify that study samples of particular types clustered with similar types from other microbial studies, we estimated the UniFrac phylogenetic distance between samples and visualized the distance of variation of our current project in reference to samples from the Earth Microbiome Project. For significance testing based on distances from sequencing data, a permutation test was used. This was chosen since univariate statistical tests often assume that observations are independently and identically distributed, which is not the case with distance calculations. Similar to PERMANOVA, the group labels were shuffled, and a Kruskal-Wallis test was applied. P values were calculated by (#(K > Kp) + 1)/(number of permutations + 1) where K is the Kruskal-Wallis statistic on the original statistic and Kp is the Kruskal-Wallis statistic computed from the permuted grouping. One thousand permutations were used for the permutation test.

#### Longitudinal data analysis

We used Bayesian Sparse Functional Principal Components Analysis (SFPCA) [69] methodology to model temporal variations and sample type differences in viral load. To quantify the contribution of potential source environments (i.e., patient microbiome) to the hospital surface microbiome (as a sink), SourceTracker2 [31] was used.

#### Random forest analysis

We performed machine learning analysis of bacterial profiles derived from 16S rRNA gene amplicon sequencing from multiple sample types (nares, skin, stool, inside floor, outside floor, and bed rail) to predict the samples’ SARS-CoV-2 status according to RT-qPCR results (i.e., “positive” or ”not detected”). For each sample type, a random forest sample classifier was trained based on the ASV-level bacterial profiles with tuned hyperparameters as 20-time repeated, stratified 5-fold cross-validation using the R caret package [70]. The dataset of each sample type was repeatedly split into five groups with similar class distributions, and we trained the classifier on 80% of the data and made predictions on the remaining 20% of the data in each fold iteration. We evaluated each classifier using both area under the receiver operating characteristic curve (AUROC) and area under the precision-recall curve (AUPRC) based on the samples’ predictions in the holdout test set using the R PRROC package [71]. For all six sample types, our data had an imbalanced representation of SARS-CoV-2 status, and “not detected” was consistently the majority class (nares: 45 not detected vs. 31 positives; forehead skin: 63 not detected vs. 16 positives; stool: 33 not detected vs. 11 positives; inside floor: 67 not detected vs. 40 positive; outside floor: 81 not detected vs. 27 positives; bed rail: 38 not detected vs. 8 positives). To assess how well a classifier can predict the SARS-CoV-2 positive samples (the minority class) using microbiome data, the AUPRC was calculated by assigning “positive” as the positive class. Next, the importance of each ASV for the prediction performance of the four classifiers with AUROC ≥ 0.7 and AUPRC ≥ 0.6 (for nares, forehead skin, stool, and inside floor) was estimated by the built-in random forest scores in the 100-fold cross-validation. For each body site or environmental site, we finally ranked all ASVs by their average ranking of importance scores in the 100 classification models. The code for generating the multi-dataset machine learning analysis is available at https://github.com/shihuang047/crossRanger and is based on random forest implementation from R ranger package [72].

To identify the ASVs consistently important to the prediction of SARS-CoV-2 across the four well-performing classifiers of four different sample types, we visualized the top 100 ranked important ASV’s and their phylogenetic relationship for each sample type using EMPress [35].

#### Redundancy analysis

To quantify the effect size of different metadata variables on our 16S rRNA gene amplicon sequencing dataset, we applied redundancy analysis on the robust Aitchison principal coordinates analysis biplot [73] as described previously [33]. Briefly, RDA employs the *varpart* function in R which uses linear constrained ordination to estimate the independent and shared contributions of multiple covariates on microbiome composition variation.

## Supplementary Information

**Additional file 1: Figure S1.** Patient (n = 16) demographics (A), antibiotics intake (B), comorbidities (C).

**Additional file 2: Figure S2.** Ili’ spatial mapping of standard hospital (non-ICU) room and intensive care unit (ICU) room. Heatmap depicts the percent of samples collected at each site that were positive for SARS-CoV-2.

**Additional file 3: Figure S3.** Snapshot of variability in longitudinal sample collection and SARS-CoV-2 viral RNA load per swab between patients and their hospital rooms, starting at patient admission time. For samples where SARS-CoV-2 was detected (+), a darker color indicates a higher viral load. White boxes represent samples with no detectable virus (-). Patient A was admitted 12 days after symptom onset and was moved to a general surgery unit room after 6 days in the ICU. Patient B was admitted 8 days after symptom onset and moved from general surgery to the ICU, where they were intubated. Patient C was admitted to the ICU 9 days after symptom onset, and despite having symptoms consistent with COVID-19 repeatedly tested negative by clinical nasopharyngeal swab; their only clinical positive came from a tracheal aspirate sample mid-way through their stay in the ICU.

**Additional file 4: Figure S4.** Source tracker on meta-analysis data. Floor samples formed a distinct cluster in this dataset; source tracking [31] with floor samples (n = 215) as the sink and meta-analysis samples (n = 1,990) as the source reveals that these floor samples match other built environment samples. The other built environment samples included in this meta-analysis were mostly floor (27.7%), faucet handles (19.6%), and gloves (15%).

**Additional file 5: Figure S5.** Beta diversity has a statistically significant but weak correlation with viral load. PCoA of unweighted UniFrac distances between samples, with SARS-CoV-2 positive samples colored by viral load across the whole dataset (A) and subset by each patient with at least one surface positive (B). Statistical analysis performed with Adonis (PERMANOVA) found a small (R^2^ < 0.01) but significant (*p-value* = 0.043) association between beta diversity and viral load across all samples.

**Additional file 6: Figure S6.** Floor sample SARS-CoV-2 status is associated with higher biomass and with significantly different bacterial community composition. Two independent metrics were used to assess biomass; 16S rRNA gene amplicon sequencing read count, which because of our equal volume sequencing library pooling approach correlates with total bacterial load [27, 74], and the Ct value from the CDC’s human RNAse P RT-qPCR target, which correlates with human biomass. (A) Abundance of 16S rRNA gene amplicon sequencing read count in SARS-CoV-2 positive floor samples showing no correlation with SARS-CoV-2 viral load. (B) Ct value of human RNAse P in SARS-CoV-2 positive floor samples showing significant correlation with SARS-CoV-2 viral load. Statistical analysis of scatter plots represents Pearson correlation, and box plots represents independent t-tests; *p < 0.05, **p < 0.01, ***p < 0.001. The legend in panel B applies to panel A as well. (C) Effect size of significant, non-redundant variables identified from Redundancy Analysis on unweighted UniFrac PCoA of floor samples.

**Additional file 7: Figure S7.** Bacterial (16S rRNA gene amplicon sequencing read count) and human biomass (RNAse P Ct) is higher in floor samples than other surface sample types.

**Additional file 8: Figure S8.** Random Forest classifier performance with 100-fold cross validation in the outside floor (n = 108; 81 not detected vs. 27 positives) and bed rail samples (n = 46; 38 not detected vs. 8 positives).

**Additional file 9: Figure S9.** Unweighted UniFrac distance between forehead and nares samples from the same host. ‘Shedder’ (n = 12) is a patient who had detectable virus on the surface in their room and ‘non-shedder’ (n = 4) did not. Bootstrapped Kruskal-Wallis p-value is 0.003.

**Additional file 10: Table S1.** Hospital surface materials and cleaning practices.

**Additional file 11: Table S2.** Top 100 random forest importance ranks and GreenGenes taxonomy from nares, forehead, stool, and inside floor samples.

## Data Availability

The dataset supporting the conclusions of this article is available in the European Bioinformatics Institute repository [ERP124721, https://www.ebi.ac.uk/ena/browser/view/PRJEB41002]. Additionally, sequencing data and processed tables and taxonomy assignments are available through QIITA [67] under study ID 13092.
