# Supplementary figures and images for: SARS-CoV-2 detection status associates with bacterial community composition in patients and the hospital environment
Source: Microbiome. 2021 Jun 8;9:132. doi: 10.1186/s40168-021-01083-0 (PMC8186369; doi:10.1186/s40168-021-01083-0)

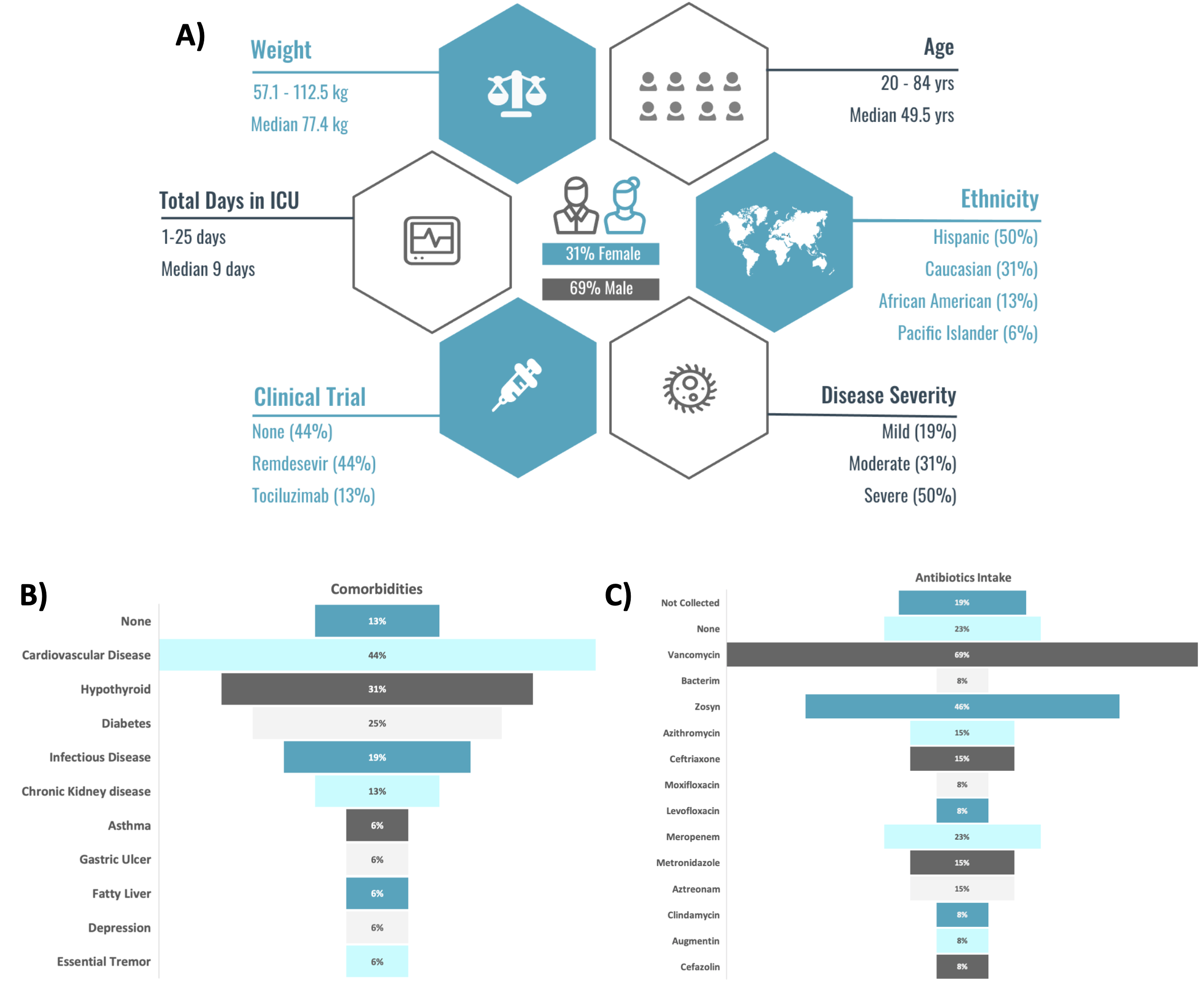

Supplement: Supplementary file 2 — Additional file 1: Figure S1. Patient (n = 16) demographics (A), antibiotics intake (B), comorbidities (C). [file 40168_2021_1083_MOESM2_ESM.docx]

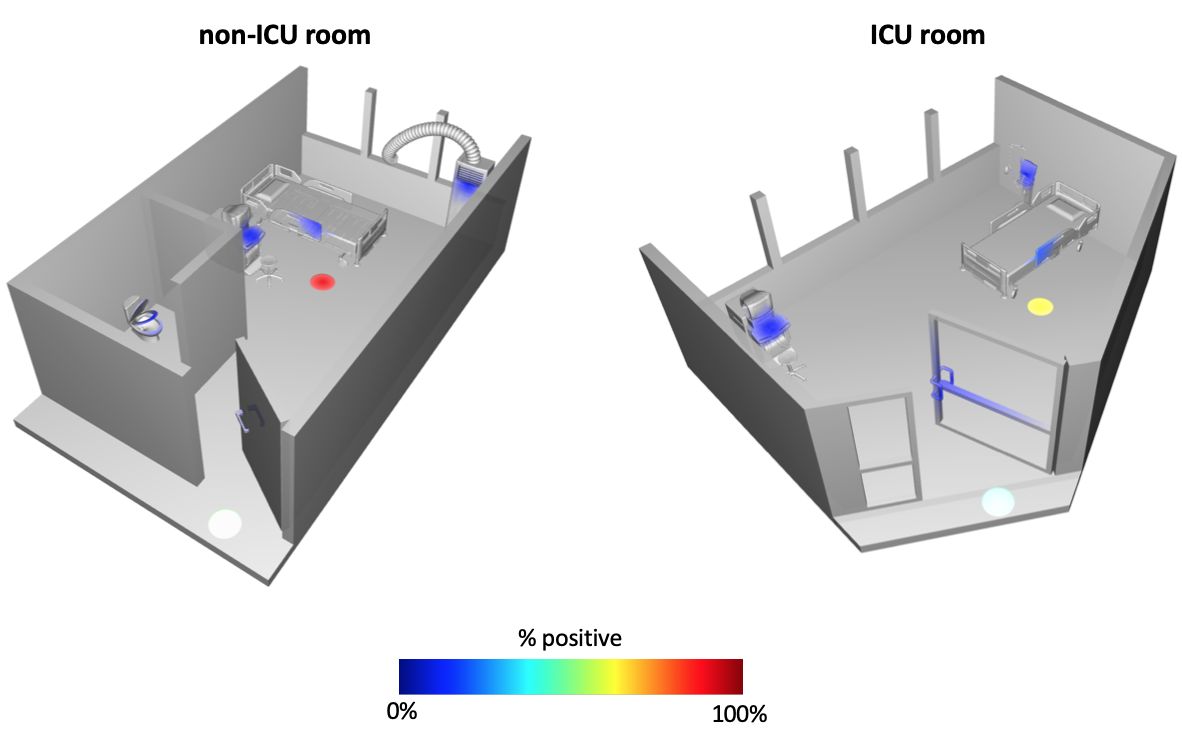

Supplement: Supplementary file 3 — Additional file 2: Figure S2. Ili’ spatial mapping of standard hospital (non-ICU) room and intensive care unit (ICU) room. Heatmap depicts the percent of samples collected at each site that were positive for SARS-CoV-2. [file 40168_2021_1083_MOESM3_ESM.docx]

**
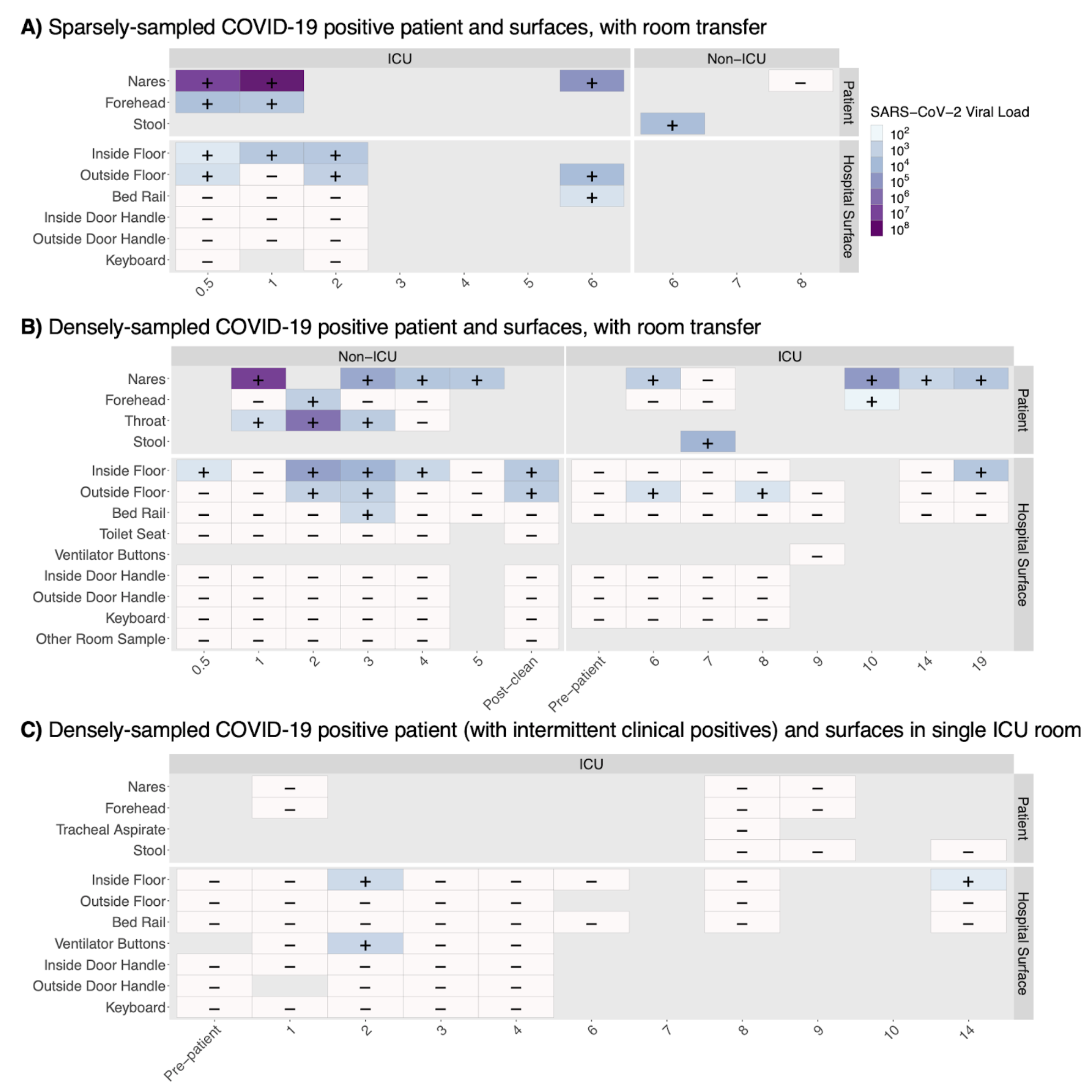
**

Supplement: Supplementary file 4 — Additional file 3: Figure S3. Snapshot of variability in longitudinal sample collection and SARS-CoV-2 viral RNA load per swab between patients and their hospital rooms, starting at patient admission time. For samples where SARS-CoV-2 was detected (+), a darker color indicates a higher viral load. White boxes represent samples with no detectable virus (-). Patient A was admitted 12 days after symptom onset and was moved to a general surgery unit room after 6 days in the ICU. Patient B was admitted 8 days after symptom onset and moved from general surgery to the ICU, where they were intubated. Patient C was admitted to the ICU 9 days after symptom onset, and despite having symptoms consistent with COVID-19 repeatedly tested negative by clinical nasopharyngeal swab; their only clinical positive came from a tracheal aspirate sample mid-way through their stay in the ICU. [file 40168_2021_1083_MOESM4_ESM.docx]

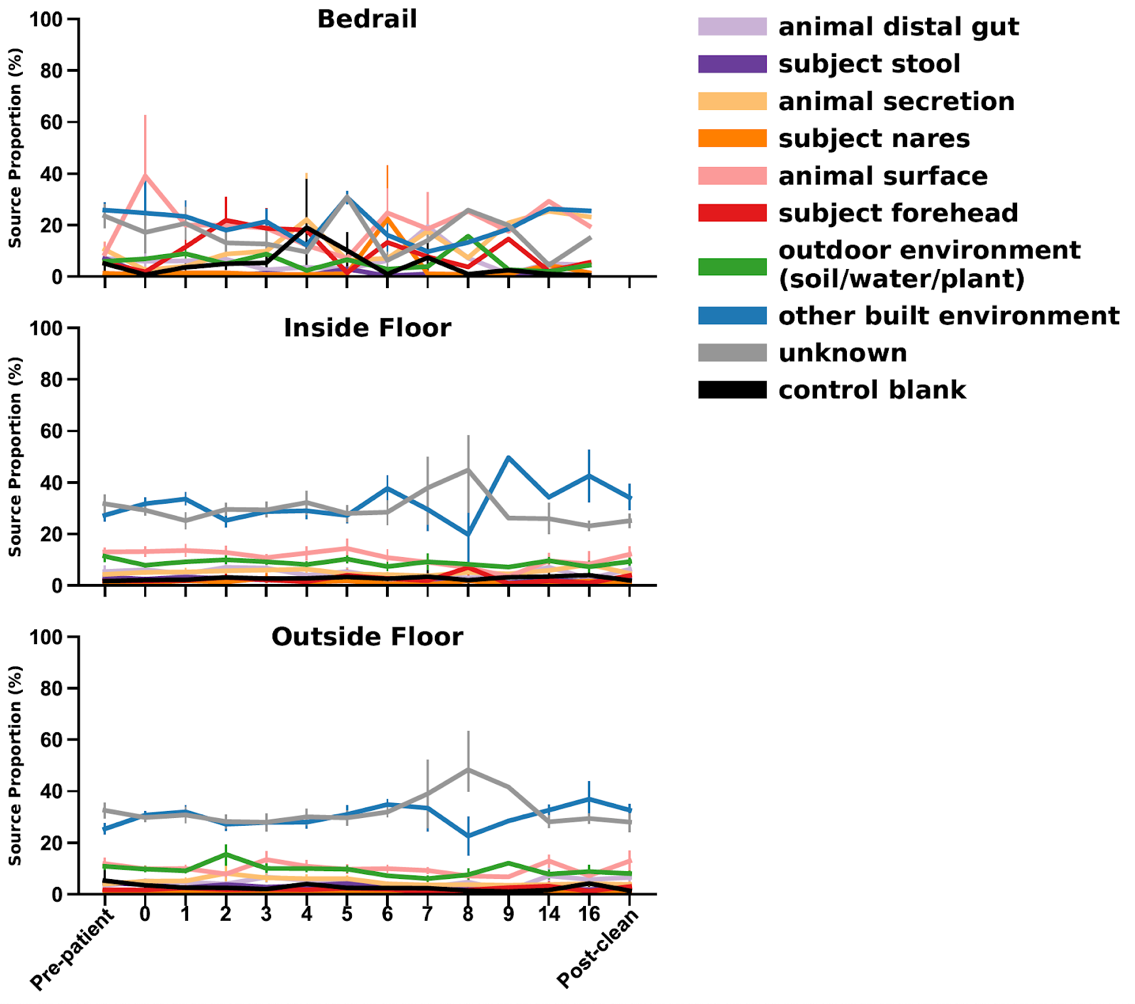

Supplement: Supplementary file 5 — Additional file 4: Figure S4. Source tracker on meta-analysis data. Floor samples formed a distinct cluster in this dataset; source tracking [31] with floor samples (n = 215) as the sink and meta-analysis samples (n = 1,990) as the source reveals that these floor samples match other built environment samples. The other built environment samples included in this meta-analysis were mostly floor (27.7%), faucet handles (19.6%), and gloves (15%). [file 40168_2021_1083_MOESM5_ESM.docx]

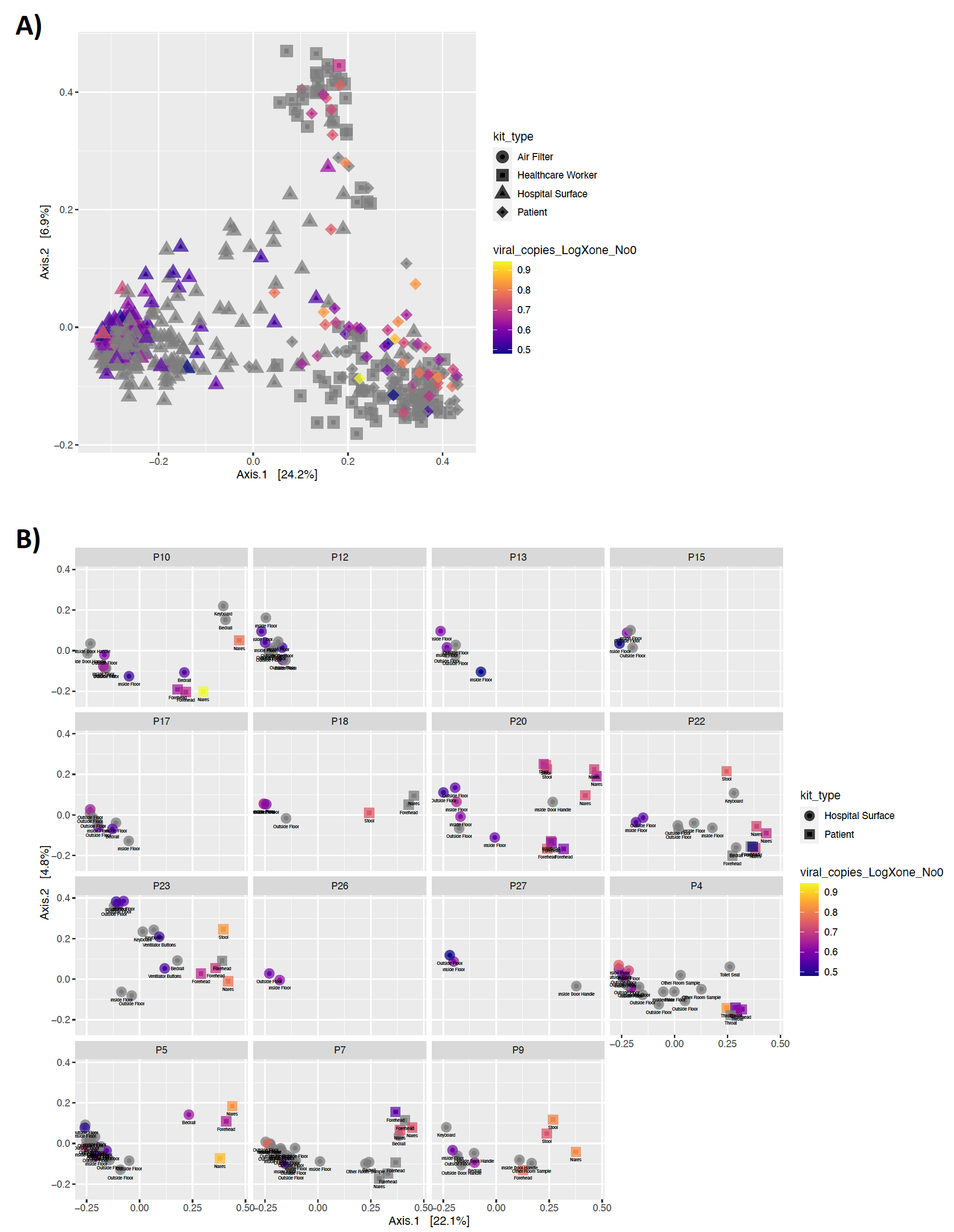

Supplement: Supplementary file 6 — Additional file 5: Figure S5. Beta diversity has a statistically significant but weak correlation with viral load. PCoA of unweighted UniFrac distances between samples, with SARS-CoV-2 positive samples colored by viral load across the whole dataset (A) and subset by each patient with at least one surface positive (B). Statistical analysis performed with Adonis (PERMANOVA) found a small (R2 < 0.01) but significant (p-value = 0.043) association between beta diversity and viral load across all samples. [file 40168_2021_1083_MOESM6_ESM.docx]

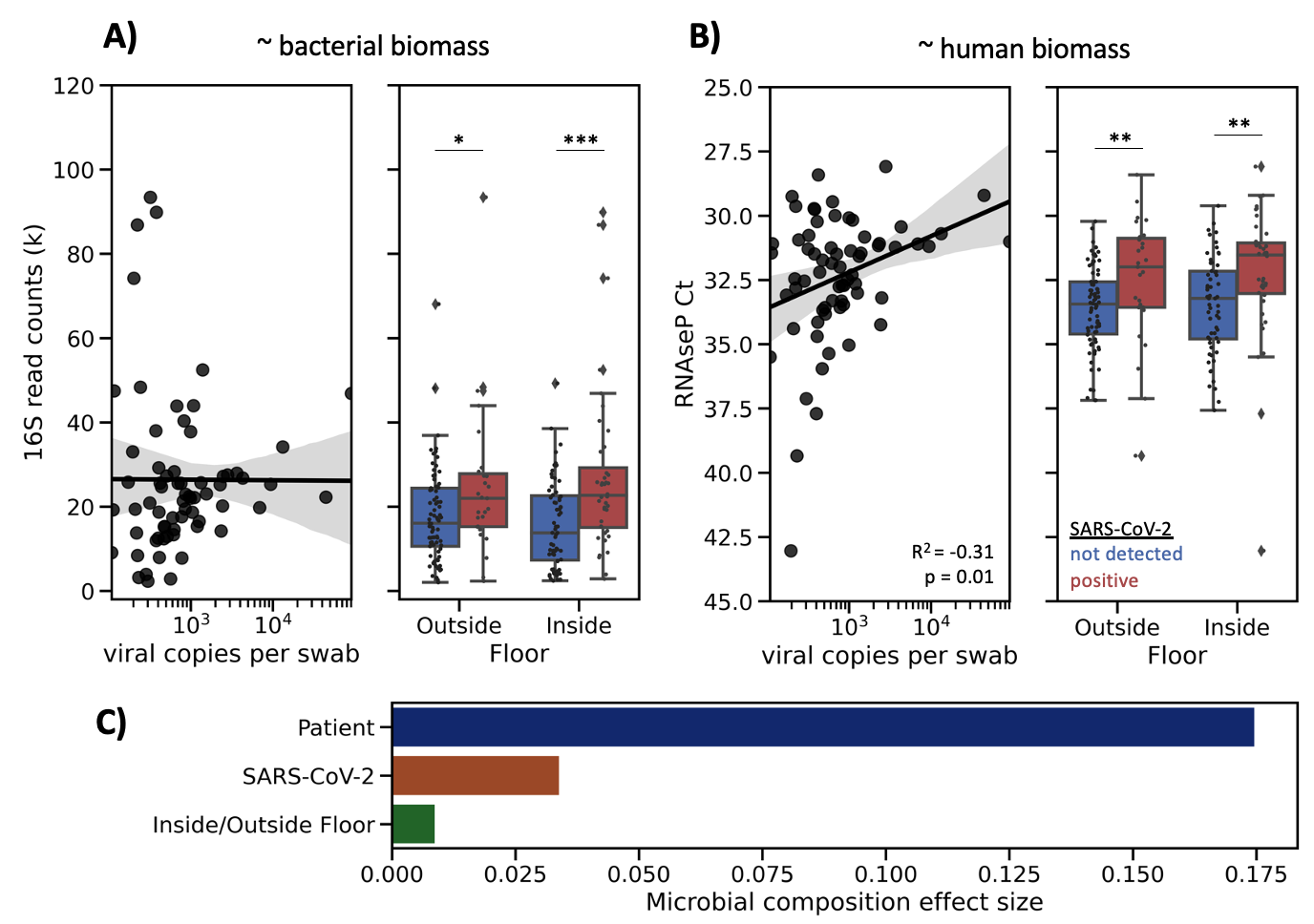

Supplement: Supplementary file 7 — Additional file 6: Figure S6. Floor sample SARS-CoV-2 status is associated with higher biomass and with significantly different bacterial community composition. Two independent metrics were used to assess biomass; 16S rRNA gene amplicon sequencing read count, which because of our equal volume sequencing library pooling approach correlates with total bacterial load [27, 74], and the Ct value from the CDC’s human RNAse P RT-qPCR target, which correlates with human biomass. (A) Abundance of 16S rRNA gene amplicon sequencing read count in SARS-CoV-2 positive floor samples showing no correlation with SARS-CoV-2 viral load. (B) Ct value of human RNAse P in SARS-CoV-2 positive floor samples showing significant correlation with SARS-CoV-2 viral load. Statistical analysis of scatter plots represents Pearson correlation, and box plots represents independent t-tests; *p < 0.05, **p < 0.01, ***p < 0.001. The legend in panel B applies to panel A as well. (C) Effect size of significant, non-redundant variables identified from Redundancy Analysis on unweighted UniFrac PCoA of floor samples. [file 40168_2021_1083_MOESM7_ESM.docx]

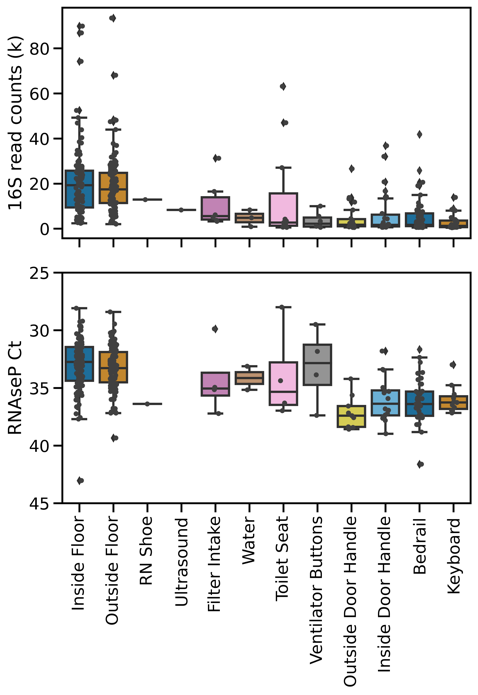

Supplement: Supplementary file 8 — Additional file 7: Figure S7. Bacterial (16S rRNA gene amplicon sequencing read count) and human biomass (RNAse P Ct) is higher in floor samples than other surface sample types. [file 40168_2021_1083_MOESM8_ESM.docx]

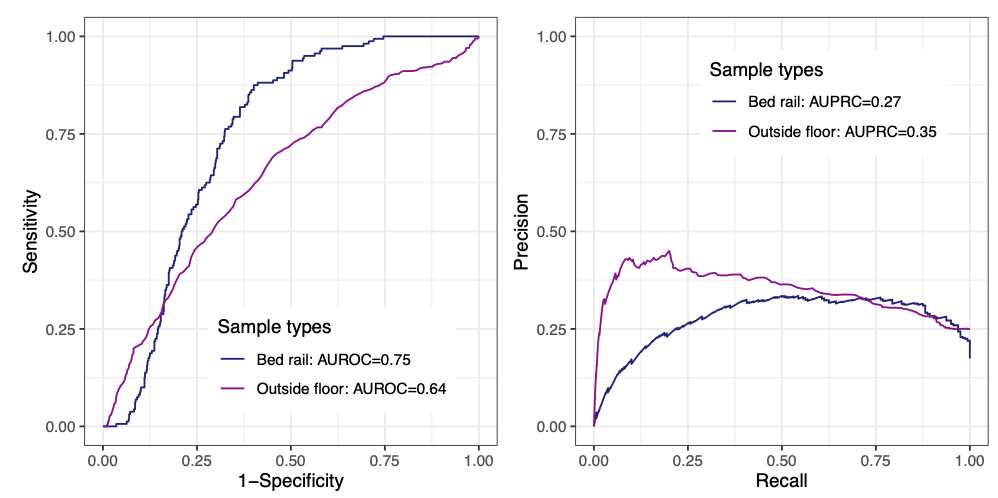

Supplement: Supplementary file 9 — Additional file 8: Figure S8. Random Forest classifier performance with 100-fold cross validation in the outside floor (n = 108; 81 not detected vs. 27 positives) and bed rail samples (n = 46; 38 not detected vs. 8 positives). [file 40168_2021_1083_MOESM9_ESM.docx]

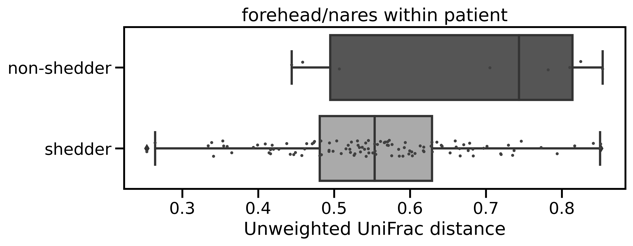

Supplement: Supplementary file 10 — Additional file 9: Figure S9. Unweighted UniFrac distance between forehead and nares samples from the same host. ‘Shedder’ (n = 12) is a patient who had detectable virus on the surface in their room and ‘non-shedder’ (n = 4) did not. Bootstrapped Kruskal-Wallis p-value is 0.003. [file 40168_2021_1083_MOESM10_ESM.docx]

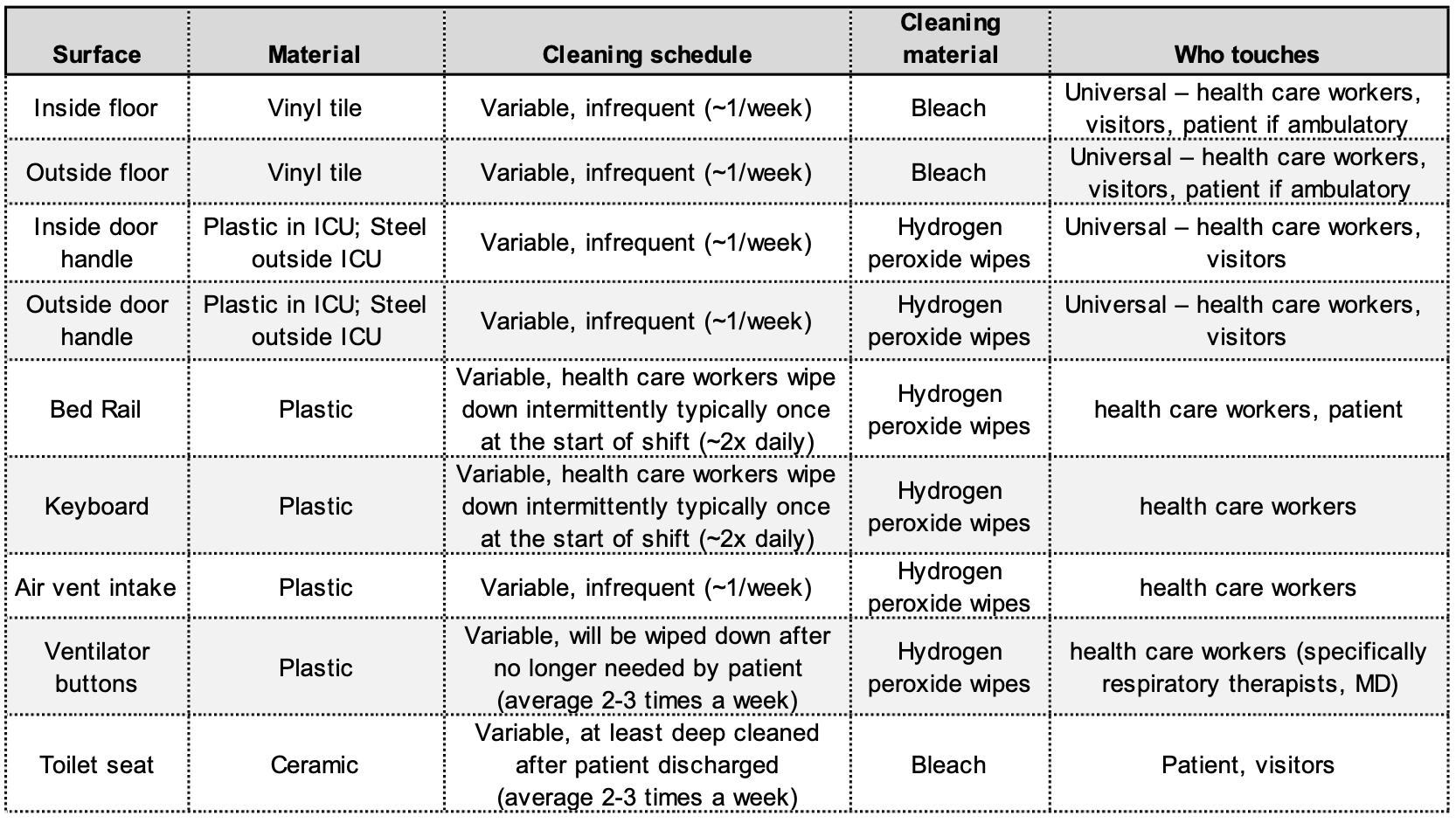

Supplement: Supplementary file 11 — Additional file 10: Table S1. Hospital surface materials and cleaning practices. [file 40168_2021_1083_MOESM11_ESM.docx]
